# Design and experimental testing of a force-augmenting exoskeleton for the human hand

**DOI:** 10.1186/s12984-022-00997-6

**Published:** 2022-02-21

**Authors:** Emily R. Triolo, Brett F. BuSha

**Affiliations:** 1 Department of Biomedical Engineering, The College of New Jersey, 2000 Pennington Road, STEM Building, Ewing, NJ 08628 USA; 2 Department of Mechanical Engineering, University of Washington, Seattle, WA 98195 USA

**Keywords:** Exoskeleton device, Medical robotics, Rapid prototyping

## Abstract

**Background:**

Many older Americans suffer from long-term upper limb dysfunction, decreased grip strength, and/or a reduced ability to hold objects due to injuries and a variety of age-related illnesses. The objective of this study was to design and build a five-fingered powered assistive exoskeleton for the human hand, and to validate its ability to augment the gripping and pinching efforts of the wearer and assist in performing ADLs.

**Methods:**

The exoskeleton device was designed using CAD software and 3-D printed in ABS. Each finger’s movement efforts were individually monitored by a force sensing resistor at each fingertip, and proportionally augmented via the microcontroller-based control scheme, linear actuators, and rigid exoskeleton structure. The force production of the device and the force augmenting capability were assessed on ten healthy individuals with one 5-digit grasping test, three pinching tests, and two functional tests.

**Results:**

Use of the device significantly decreased the forearm muscle activity necessary to maintain a grasping effort (67%, p < 0.001), the larger of two pinching efforts (30%, p < 0.05), and the palmer pinching effort (67%, p < 0.001); however, no benefit by wearing the device was identified while maintaining a minimal pinching effort or attempting one of the functional tests.

**Conclusion:**

The exoskeleton device allowed subjects to maintain independent control of each digit, and while wearing the exoskeleton, in both the unpowered and powered states, subjects were able to grasp, hold, and move objects such as a water bottle, bag, smartphone, or dry-erase marker.

## Introduction

In general, grip strength has been seen to gradually decline between 60 to 75 years of age- this decline more drastically noted among men [1]. Additionally, approximately 795,000 Americans suffer from a stroke, the leading cause of serious long-term disability, per year, reducing mobility, including upper limb dysfunction, in over half of stroke victims age 65 and older [2]. Upper limb dysfunction, including decreased grip strength and/or diminished ability to hold objects is also prevalent in populations with carpal tunnel syndrome [3].

Robotic exoskeleton devices can be primarily designed to augment user strength in order to assist with activities of daily living (ADLs), or as rehabilitative devices that are used under the guidance of a physical therapist to help patients regain greater functionality of damaged joints and/or muscles [4]. Assistive exoskeletons for the hand can be grouped according to how the augmenting forces enhance the concentric movement of the digits. Devices have been designed to apply the augmenting forces to the dorsal aspect of the fingers via mechanical linkages [5–8] or fabric-based pneumatic bladders [9]. A ventral approach has also been used, where pseudo-tendons applied tension that is transmitted to the digits through soft [10] or hard exoskeleton structures [11]. Heo, et al. [12] and Bos, et al*.* [13] have both published comprehensive listings and reviews of exoskeleton devices for the hand.

Regardless of the technique used to apply the augmenting force, for an assistive device to function, finger movement or another indication of the user’s intent to move must be sensed and transformed into a signal that controls the application of the assistive forces. Ideally, there needs to be a consistent coordination between the device and the user that results in a coupling of the human hand and the augmenting system, allowing the robotic device to consistently provide assistance as needed through the detection and amplification of the user’s effort. Some grip-assistive devices, however, have pre-programed algorithms with which users do not initiate by intent to move. These types of devices, such as the HERO Grip Glove [14] move the user’s hand through gripping and/or pinching patterns that allow for a set force production, which is then augmented by a user’s own strength. Devices by Yap et al*.* and Polygerinos et al. operate in a similar fashion, where the user shows intent to move, and the device then moves through a pre-determined motion without any subsequent input from a user [15, 16]. Such devices can both be used for hand motion training with the guidance of a physical therapist, as well as assist in ADLs.

Hand exoskeleton designs vary in overall weight, complexity, and cost. In attempts to provide the full range of motion of the human hand to the user, most of these devices have become both bulky and complex, and due to this are restricted to a single functional activity- either hand-opening or pinching. These exoskeleton devices often use a single motor or driving feature to assist multiple fingers [14], such as with Yoo et al.’s design which used one motor to drive three fingers [17] and Gasser et al.’s design which uses two motors to control four fingers [18]. Alternatively, some devices actively assist fewer than all five fingers [19], for example, Pu et al*.*’s, Nycz et al.’s, and Gasser et al*.*’s designs exclude the thumb [6, 18, 20]. Devices that allow for more degrees of freedom and independently assist all five digits become exceedingly cost prohibitive as more joints, motors, and custom electrical components become necessary [5, 21]. These additional motors and therefore batteries will also make the device heavier and potentially tethered to a power source dependent on the current draw [9, 22].

A previous design from our laboratory used machined aluminum segments to construct exoskeleton digits with a desktop computer-based control system tethered to the device [23, 24]. In order to reduce both the manufacturing cost and time of these previous prototypes as well as the weight, the most recent exoskeletons were designed to be constructed with 3-D printed thermoplastics. Furthermore, a minicomputer-based control system replaced the desktop computer and associated data acquisition hardware, which provided a further reduction in cost, weight, and complexity, and also allowed for greater freedom of movement [11, 25]. Even though exoskeletons with 1 or 2 fingers are simpler to implement, most ADLs require at least 3 fingers to be assisted by an exoskeleton [26]. Exoskeletons with 3 or 4 fingers could assist with most ADLs, the realism for the user would decrease as the number of fingers decrease [26]. Additionally, having fewer fingers limits the grasping positions the user can make with their hand, as well as limit the objects the user can lift. For example, a 4 or 3-fingered exoskeleton could assist a user in picking up objects of uniform shapes (e.g., cup, reusable water bottle) but not objects that are oddly shapes or have varying thicknesses (e.g., cell phone, wine glass). Increasing the number of independently controlled exoskeleton digits would allow for the control to lift objects such as this and increase the mobility to the point where it feels natural to the user. For training and rehabilitation devices specifically, being able to independently control each finger is imperative for re-developing muscle and flexibility in every finger. All these reasons listed are why most modern powered exoskeletons for the human hand use a five-fingered design despite the added complexity and weight [15, 16, 22, 27–29], and why we have decided to move from a three-fingered design [11, 25, 30] to a five-fingered design [31].

The main objective of this study was to design and produce a wearable powered exoskeleton for the human hand to improve structural stability of the fingers while also augmenting pinching and grasping efforts, and to validate that the device augments both the user’s pinching and grasping efforts and ability to perform ADLs by evaluating healthy human subjects. The exoskeleton device should be user friendly, allow for individual finger movement, and be cost optimized. This device aims to not compromise cost and weight for individual, independent movement of all five fingers. To be user friendly, the device must be able to incorporate a range of sizes that users may experience on a daily basis, as well as have a minimal user interface, and be easily donned and doffed. Additionally, the device must be portable and easily carried, and the batteries should last multiple hours. For cost optimization, electronic components must be commercially available, and the device should be modular such that broken parts are able to be replaced as necessary. Additionally, the modularity of the design must be such that different sized pieces are able be added and removed for users of differing size in the future. The exoskeleton structure was designed using CAD (computer aided design) software to enclose all five fingers of the right hand and was 3-D printed in ABS plastic. Each finger’s movement efforts were individually monitored and proportionally augmented via the microcontroller-based control scheme, linear actuators, and rigid exoskeleton structure.

## Materials and methods

### Mechanical structure design

**Fig. 1:**
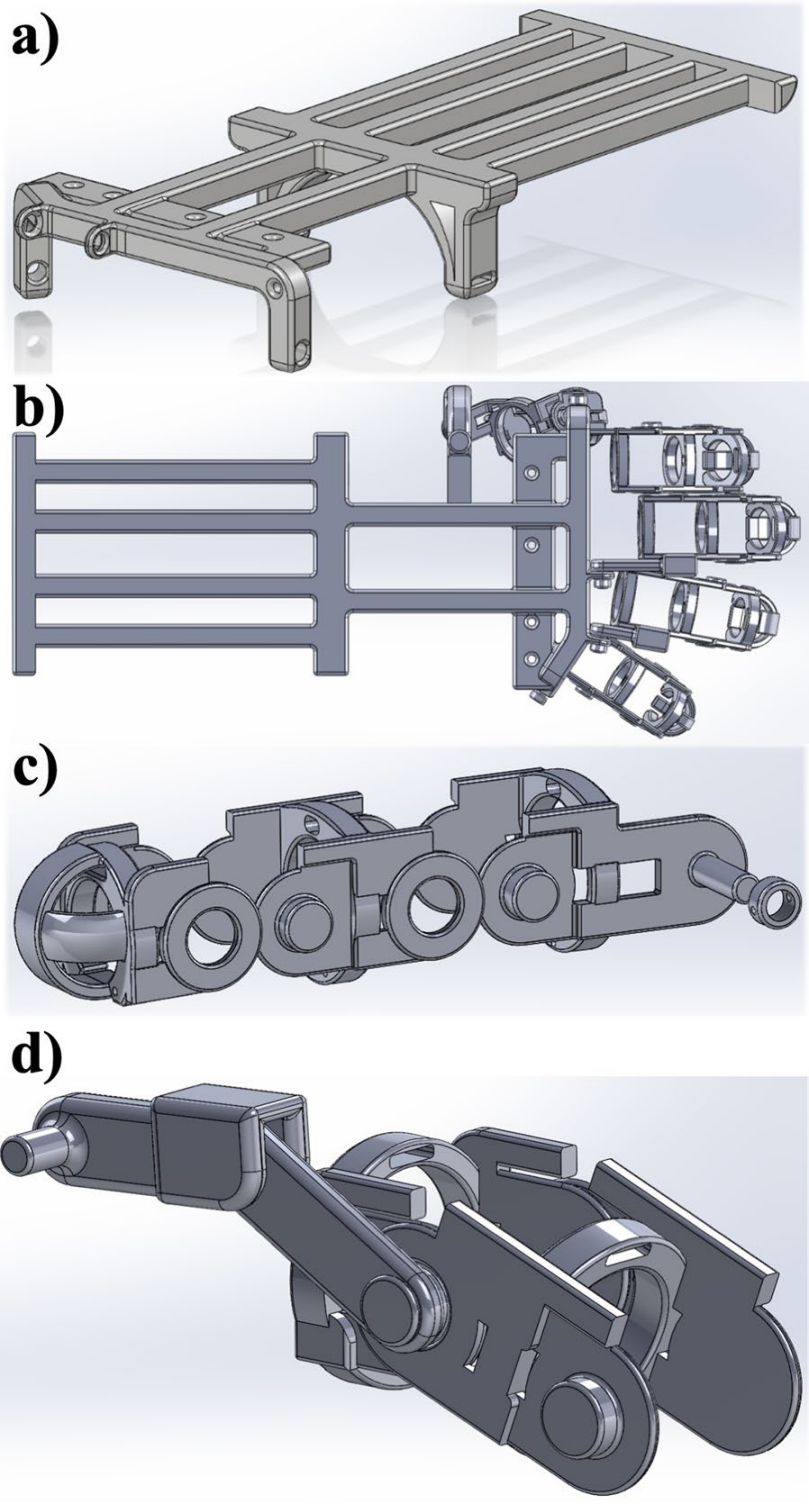
CAD rendering of **a** the exoskeleton wrist-forearm structure, which provided a secure mounting for the electrical components and Velcro wrist straps. The design provided attachment points for the five exoskeleton digits and was 3-D printed as a single piece. **b** Full hand assembly including all of the exoskeleton digits, the thumb assembly, and wrist-forearm structure. **c** The index digit exoskeleton and pinky digit were designed with similar concentric ring structures with bilateral joints. The distal sections of each finger assembly encase the fingertips, and the section proximal to the wrist and forearm structure attached to the corresponding wrist-forearm attachment point with a pin and securing cap. Individual sections were 3-D printed separately using ABS. **d** The ring digit exoskeleton flexible knuckle joint and middle digit knuckle joint, which is constructed in the same manner, were 3-D printed as shown, with the distal cap of the same design to the index and pinky digits 3-D printed and hand-assembled

The mechanical structure of the device was designed as three components: an exoskeleton that surrounded and supported the movement of each of the five digits of the hand; a rigid wrist-forearm structure that attached to the forearm and prevented any movement of the wrist; and a laser-cut acrylic box attached to the dorsal aspect of the forearm structure that contained the control system, electric motors, and batteries. The exoskeleton digits and the wrist-forearm structures were designed in SolidWorks and 3-D printed in ABS plastic (Dimension SST 1200s). The wrist and forearm structure was designed to distribute the weight of the electrical components and motors along the dorsal aspect of the forearm, and to be easily donned and doffed using two Velcro straps, as illustrated in Fig. 1a.

The exoskeleton digits were 3-D printed individually as shown in Fig. 1c, and assembled as describe previously by Triolo et al*.* [31] (Fig. 1b) to provide powered flexion to each digit. Additionally, rubber bands were attached to the dorsal aspect of each digit to provide passive assistance in fully extending the digits and to offset friction produced by insulated electrical wires and rubbing of the individual pieces of the exoskeleton digits. The index and pinky exoskeleton digits each have 3 degrees of freedom, allowing for powered flexion and passive extension of the fingers by lining the rotational joints up with each knuckle (Fig. 1c). The ring and middle exoskeleton digits each have 4 joints but allow for 3 degrees of freedom, allowing for powered flexion and passive extension of those fingers by lining up the distal two knuckles with rotational joints and using the top two joints for proximal knuckle motion (Fig. 1d). This knuckle joint allows for non-assisted adduction and abduction. The thumb has three degrees of freedom, which allow for flexion and extension of the thumb via rotational joints lined up with the distal two knuckles and rotation via a hinge joint adjacent to the wrist-forearm structure. In total, the exoskeleton device has 15 degrees of freedom with mechanical stops on the dorsal aspect of each digit to prevent hyperextension, but allow for the full 164° range of motion, which allows able-bodied individuals to move their fingers to accomplish standard activities of daily living. The device also allowed for a 15.4 cm open hand length total (tip of exoskeleton thumb to tip of exoskeleton pinky).

The design of the device is modular, so the wrist-forearm structure was designed to allow for the exoskeleton digits to be individually attached or removed, as illustrated in Fig. 1b. The exoskeleton digits were attached to the wrist-forearm structure using a pin and cap design to allow to replacement and repair of the pieces of digits directly attached to the structure without the need to reprint the large wrist-forearm structure, reducing manufacturing time and cost. The thumb was connected directly to the wrist, with the distal component of the thumb designed to be attached after printing. The components of middle and ring digits were designed similarly, to allow for assembly after printing. This modular design also allows the digits to be replaced with smaller or larger digits in order to allow for smaller or larger hand sizes. Non-slip grip tape was placed on the tips of the exoskeleton digits to prevent scratches to both the plastic exoskeleton digits and to objects that the exoskeleton assists the user in lifting, as well as to prevent objects from slipping through the hard plastic exoskeleton digits.

### Electrical components and control system design

A 0.2″ diameter force sensing resistor (FSR) was attached to the inner ventral aspect of the each of the exoskeleton digits using small sections of Velcro to prevent damage to the FSRs. Each FSR was connected to the microcomputer control system to monitor individual finger movement via insulated wires that were routed through holes designed into the dorsal aspect of the exoskeleton structure.

**Fig. 2:**
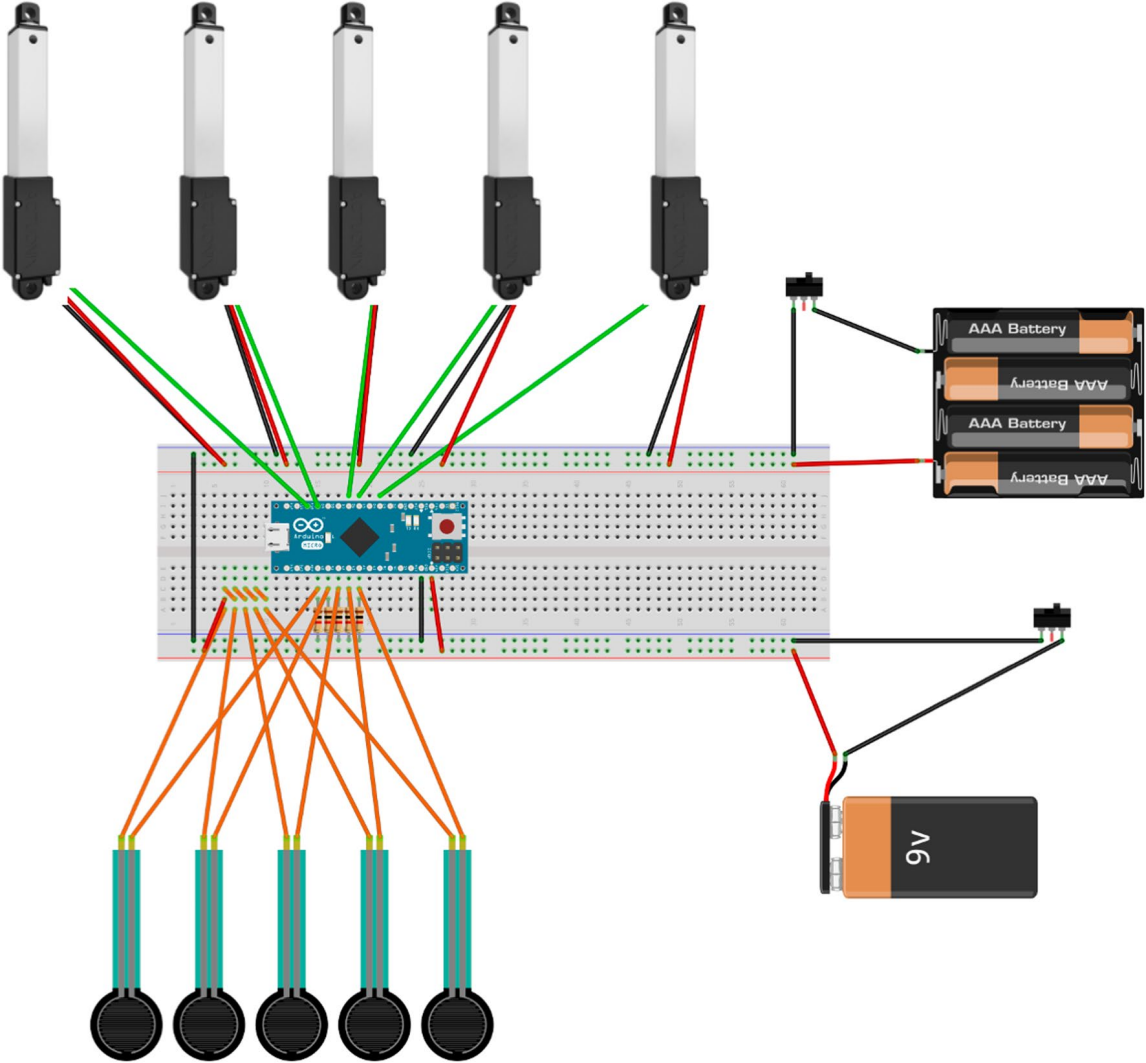
Circuity schematic for the final exoskeleton hand deign, the five servos representing the five linear actuators, the series of AAA batteries representing the 6 V rechargeable battery, and the 9 V battery representing the rechargeable 9 V battery

The five FSRs provided independent inputs to the microcontroller (Arduino micro) based control scheme, in which the FSR on each finger individually commands the corresponding linear actuator (Actuonix L12-I, 50 mm stroke length) proportional to the force provided. Each of the five linear actuators were connected to the distal exoskeleton digit fingertip via a polymer cable that was threaded though the ventral aspect of the exoskeleton digit. A 6 V Ni-MH rechargeable battery was used to provide sufficient power to the actuators, and a 9 V rechargeable battery provided power to the microcontroller and FSRs. A schematic of the control circuit is presented in Fig. 2. Using this circuitry, each linear actuator moved its full stroke length in approximately 3 s, meaning that each finger would move from fully extended to fully contracted (or vice versa) in 3 s. We have avoided using any active components to extend the fingers, as this could increase the possibility of unintentionally hyperextending the fingers to the point of causing discomfort. By using the passive elastic to extend the fingers, the device can provide enough force to assist in the extensor movement but not risk causing accidental damage. If the user were to remove the tips of their fingers from the FSRs, the exoskeleton digits would automatically extend, but would not force the extensor movement should the user resist the rubber bands, even without engaging the motors. Overall, the main purpose of the device is to augment to concentric, or grasping, efforts.

**Fig. 3:**
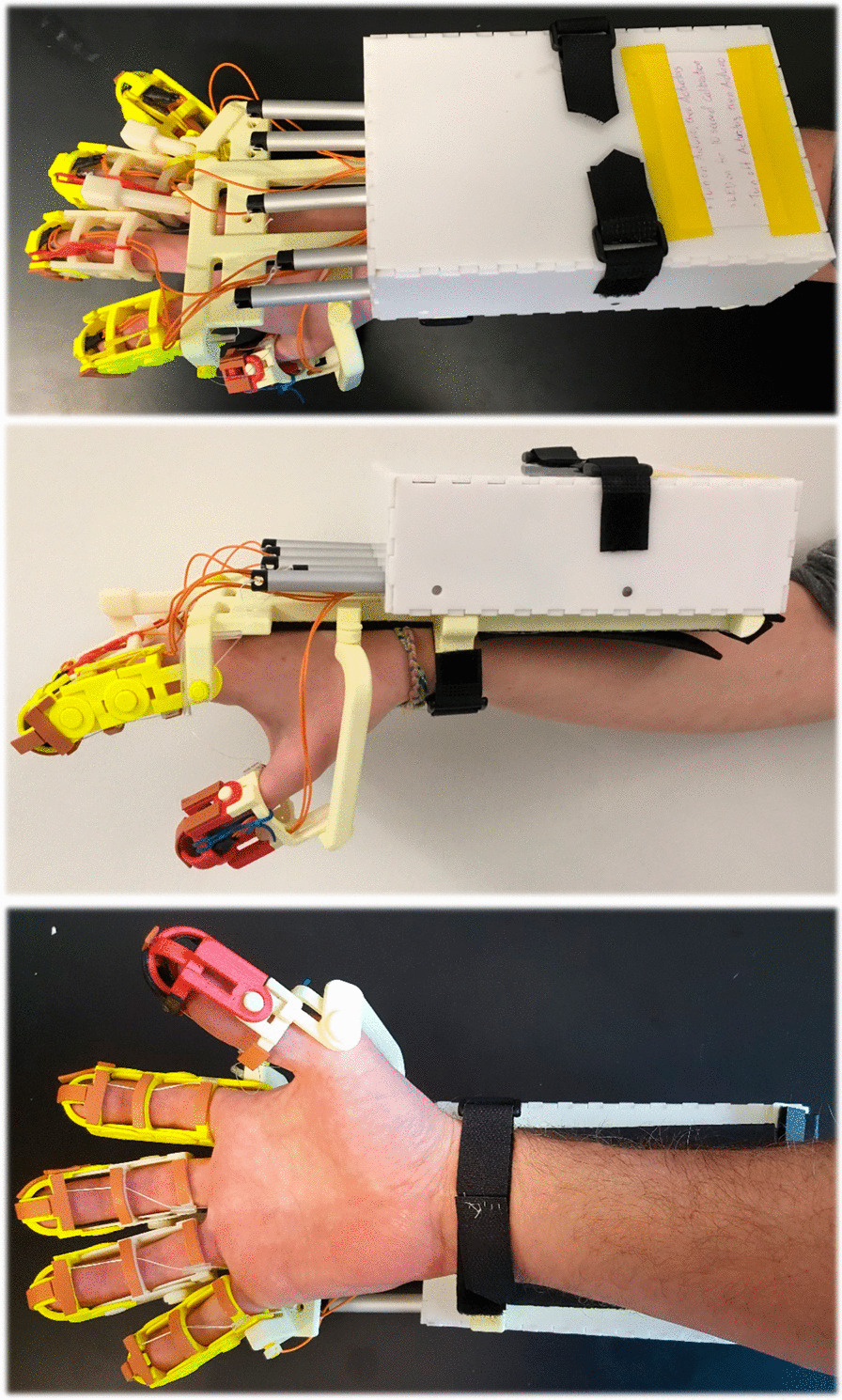
From top to bottom, a dorsal, lateral, and ventral perspective of the complete device on a user’s hand

The batteries, microcontroller, circuitry, and actuators were enclosed in a box constructed from 1/8″ thick acrylic plastic and assembled using the guidance of the laser-cut partial finger joints. The linear actuators and the wires connected to the FSRs passed through openings cut in the acrylic box facing the digits. On the side of the box, distal from the exoskeleton digits, were two switches that controlled power to the device, and an LED to indicate when the device was calibrating. The mass of the completed device, batteries included, was 0.91 kg, and the total cost of the device based on cost of parts is approximately $600. Given that every one of the motors are fully engaged half of the time the device is on, the battery life for the device before the battery must be recharged is 2.6 h. Photographs of the complete device are presented in Fig. 3.

**Fig. 4:**
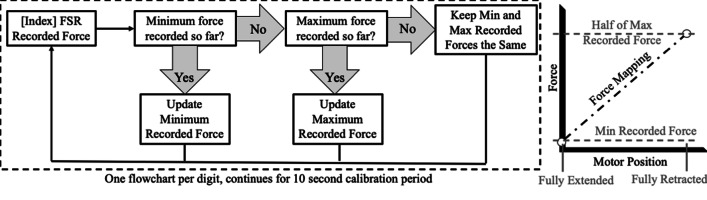
Flowchart of the 10 s calibration period, shown for the Index finger as an example. Each digit undergoes this calibration process simultaneously during the calibration period. The right plot shows the calibration curve for user force production versus motor positioning used during normal use based on the values determined during calibration

Each time the device was powered on, the device began a ten-second calibration sequence during which the motors were inactivated, indicated to the user via an illuminated LED located next to the power switches. While the device was in calibration mode, the user was instructed to make 3–5 maximal grasping efforts around a tube, and the control system independently calibrated each linear actuator to the movement of each corresponding digit, described as a flow chart in Fig. 4. The smallest pressure detected by each FSR during the calibration period was mapped to the corresponding linear actuator being fully extended (finger fully extended). Alternately, the half of the largest pressure sensed by each FSR during the calibration period was mapped to the corresponding linear actuator fully contracted (finger fully contracted). This calibration was performed by the control on a digit-by-digit basis, so each digit had its own force-position curve post-calibration (during normal use), and each actuator only moved when pressure was applied to its corresponding FSR. This allowed for precise, independent digit control, regardless of the strength of one of the user’s digits compared to their other digits.

### User-independent exoskeleton force production

**Fig. 5:**
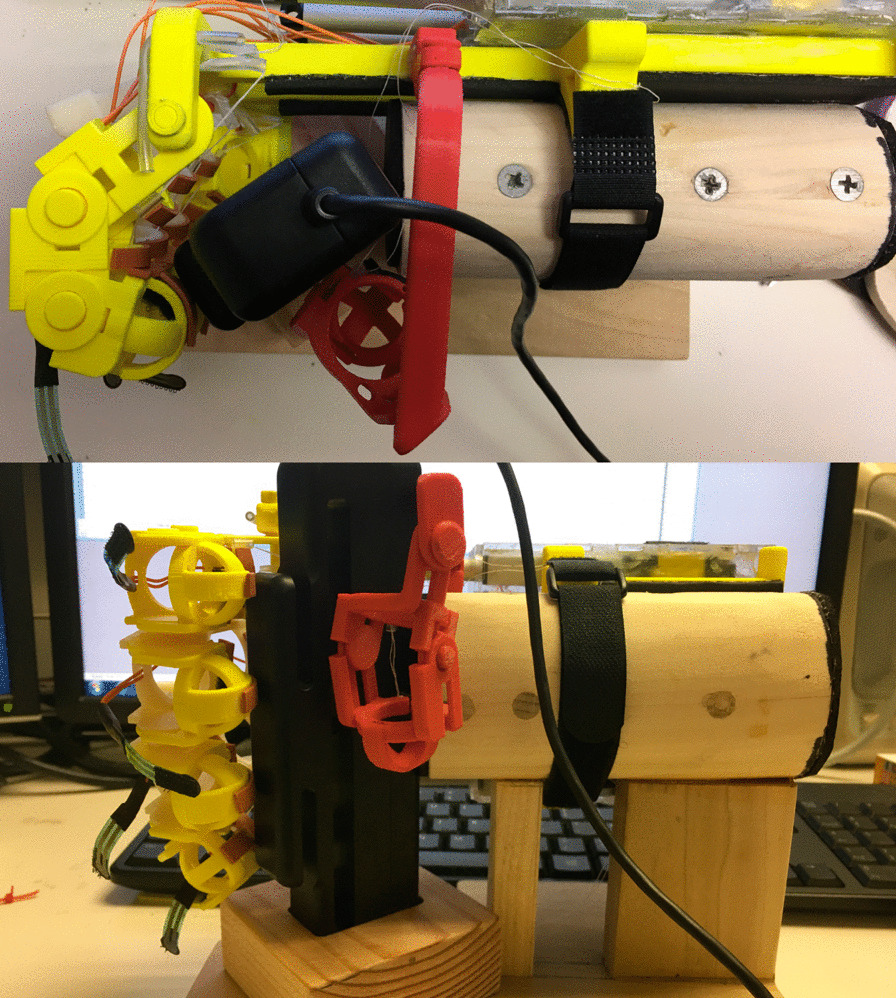
Exoskeleton hand attached to custom wooden stand, grasping around the hand force dynamometer used to determine the maximum force production of the device without human interactions

While the main focus of this effort was to reduce EMG activity per force applied (such that less muscle activation is needed to perform a task), as opposed to designing an exoskeleton to generate as great of a grasping force as possible, it is useful to fully characterize the capabilities of the device without human interaction for comparison purposes. There is no standard metric for comparing devices in terms of human use, as not all studies record EMG, so these measurements simply allow for comparisons to similar devices. To assess the exoskeleton device’s user-independent grasping and pinching force production, the unworn device was fixed into a custom wooden test-stand that allowed the digits to be positioned around a grip force dynamometer (BioPac Systems Inc.), demonstrated in Fig. 5. While affixed in the test-stand a locally designed software algorithm commanded the device to produce pinching movements to the dynamometer with the index and thumb digits, independent grasping movements against the custom stand (to mimic the palm) with the index, middle, and ring fingers independently, and a grasping force with all of the digits. For each movement, the device was commanded to produce three ten second contractions, with an un-activated rest of ten seconds between each movement.

**Fig. 6:**
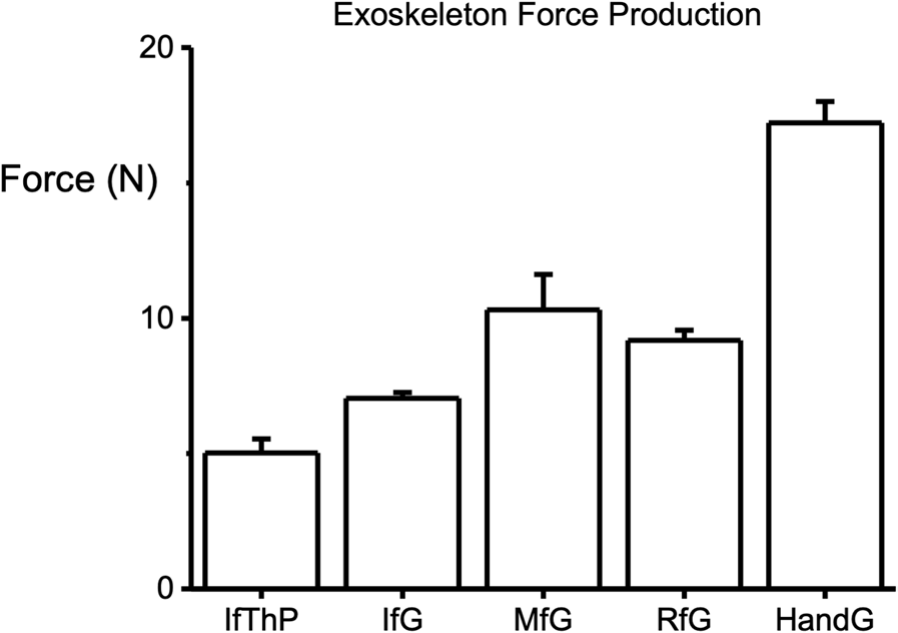
Average force production of the unworn full exoskeleton device during a pre-determined algorithm causing the device to perform multiple trials of index finger and thumb pinches (IfThP), index finger grasps (IfG), middle finger grasps (MfG), ring finger grasps (RfG), and full hand grasps (HandG)

The force measured during the un-activated phase was subtracted from the force measured during the activated phase to compensate for any baseline drift. The results of this independent force production test are shown in Fig. 6, where the maximum grasping force, during which all digits were completely contracted, was identified as 17.2 N. The smallest force produced by a unique finger configuration by the exoskeleton was the index-thumb pinch at 5.0 N. These values approximate the maximum amount of force (all motors fully engaged) that the exoskeleton is applying when assisting a user. For example, if a user is performing a grasping motion with the device powered and all of the motors are fully engaged, we can assume that the exoskeleton is assisting in 17.2 N of force during that grasp.

### Experimental methods

Ten healthy subjects with normal range of motion in the right hand, aged 18 to 23 (5 male, 5 female), participated in the study. Prior to participation, all subjects were informed of the experimental procedure, and each provided written consent. This study was approved by the Institutional Review Board of The College of New Jersey.

Prior to experimental testing, a fit test of the exoskeleton device was conducted. Each subject donned the device and determined if their hand fit comfortably, that it was possible to move all of their digits throughout the range of motion of the device, and were able to make full grasping and pinching efforts. It was also required that each of the subject’s fingers maintained contact with each corresponding FSR throughout finger movements. If necessary and on a subject-by-subject basis, the polymer cables were tightened or loosened to provide comfortable movement of each of the exoskeleton digits and to ensure that each fingertip remained in constant contact the corresponding FSR. Subjects who could not comfortably fit their hands within the exoskeleton device and/or could not complete the fit-test movements were excluded from participation.

After the fit test was concluded and it was assessed that the device was properly fit to the subject’s hand, subjects were allowed a short familiarization phase. The familiarization phase was first performed with the exoskeleton device worn, but unpowered so the subjects could become acclimated to the device. Subjects were asked to pick up several objects of varying shapes and sizes, including a water bottle, tote bag, lacrosse ball, and cell phone, as well as practice gripping and pinching maneuvers around the hand force dynamometer mentioned previously. The subjects were then asked to perform those same tasks with the exoskeleton device powered on. This familiarization phase took subjects between 5 and 15 min.

**Fig. 7:**
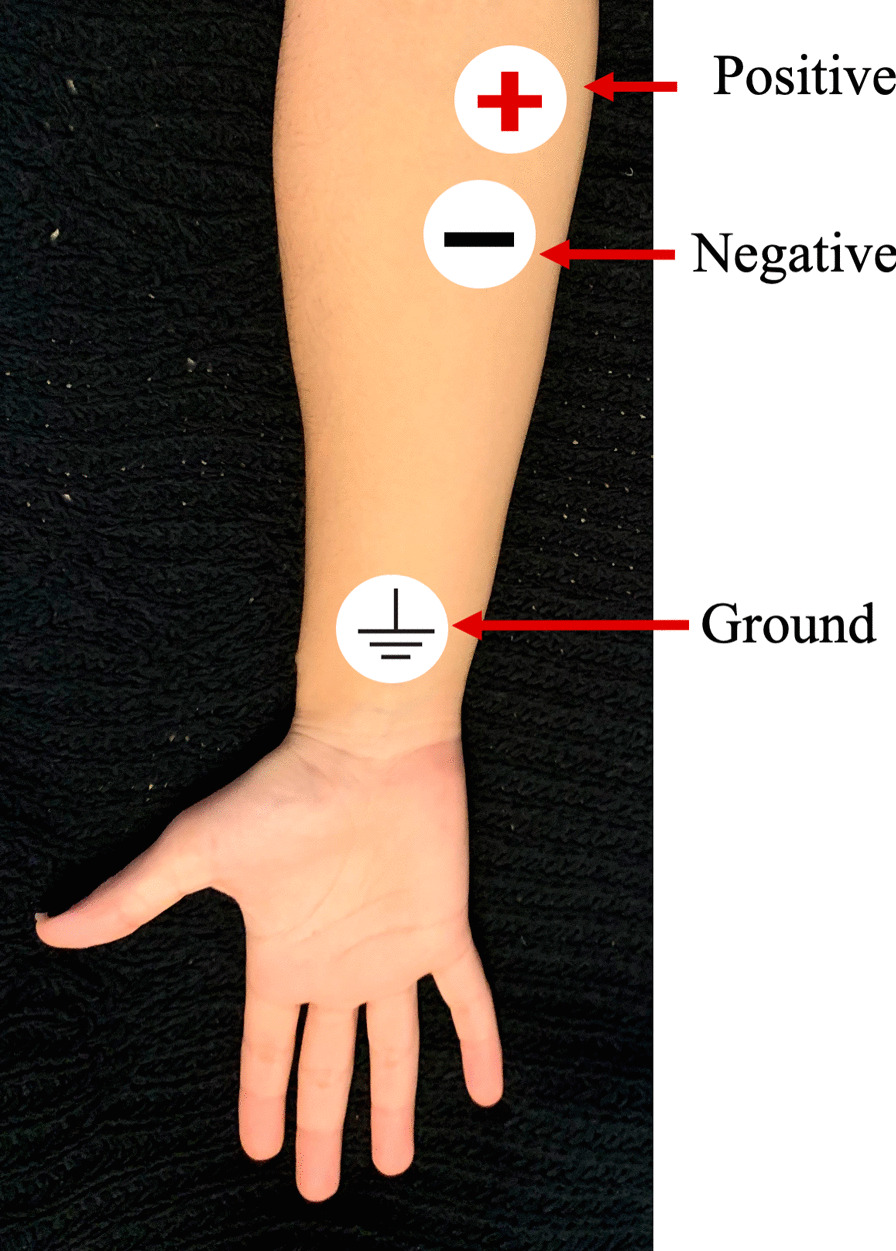
Surface EMG electrode placement on a human subject

Grasping and pinching forces produced by the users were recorded using a hand-grip dynamometer (BioPac Systems Inc.). Three surface electromyography (EMG) electrodes (Heart Trace, Cardiology Shop) were placed on the ventral aspect of the forearm proximal to the elbow to record the surface EMG of the aggregate of forearm muscles, mainly the Flexor Carpi Ulnaris, Pronator Teres, Palmaris Longus, and Flexor Carpi Radialis (Fig. 7). This simple configuration of electrodes is able to distinguish flexion due to grasping and pinching motions from extension due to opening the hand [32]. A more complex array of electrodes was not used due to both obstruction of movement of the subject during the trial and the low discrimination rate possible when observing muscle activation in the forearm due to grasping [32, 33] when compared to the additional data that would have been acquired. Additionally, activation patterns of individual muscles in the forearm have been shown to change during the same motions after a familiarization period with an exoskeleton device [34]. To assist in the visual placement of the electrodes, subjects were instructed to open and close their bare hand multiple times in order to locate the corresponding muscles by observing muscle flexing. Grasp force was low pass filtered with a cutoff at 66.5 Hz, and EMG was band pass filtered from 5 to 1000 Hz. All data were simultaneously recorded and saved with a pc-based data acquisition system (Biopac Systems, Inc.), sampled at 1 kHz.

### Experimental protocol

In order to determine whether the exoskeleton device is significantly augmenting both pinching and grasping efforts, we asked the subjects to generate and maintain a target force of approximately 10% of the average maximum for a healthy individual, and EMG measurement both bare-handed and while wearing the device were compared. By showing that the device either augments or neither augments nor impedes the ability of healthy individuals to perform ADLs, then it is more likely that using the device will assist impaired individuals in performing those same ADLs. By showing a decrease in force/EMG with a particular task, this implies that the device makes the completion of the task easier. With their arm resting on the benchtop, each subject produced three five-second 25 N grasping efforts, first while bare handed, second while wearing the unpowered exoskeleton, and finally while wearing the powered exoskeleton. The testing-states (bare handed, wearing the unpowered device, and wearing the powered device) were repeated while the subject produced: 5-s pinching efforts of 15 N with their thumb and forefinger; the same pinching effort with a force of 8 N; and 15 N pinching efforts with their thumb, forefinger, and middle finger (palmer pinch). To assist in maintaining the target forces, the subject was provided constant visual feedback on a computer monitor of the force measured by the dynamometer.

In addition to these grasping and pinching tests, two functional tests were also performed; however, only EMG was recorded and the duration of the efforts was increased to ten seconds per replicate. In the first, the subject lifted a plastic water bottle (0.5 kg) off the table with their elbow resting on the table. In the second, the subject picked up a tote bag filled with binders and papers (2.4 kg) off the floor with a straight arm. These trials were recorded with the same filtering as the previous tests. Subjects performed all of the above tasks first bare-handed, then while wearing the unpowered device, and the finally while wearing the powered device. The entire experimental protocol took subjects between 45 min to 1 h and 15 min to complete.

### Data analysis

A locally designed MATLAB algorithm was used to identify peak pinching and grasping forces per test, as well as the troughs in force between efforts (used as a baseline), and to extract 1 s of EMG and force data with the peak or trough as the midpoint. To accomplish this task, the force data were moving time averaged (MTA) at 2000 ms and zero-phase filtered to exaggerate large changes in the data before being assessed for peaks and troughs with a minimum distance between peaks set as 5000 ms. The force data in the determined 1-s peak/trough intervals were then MTA at 500 ms, and the EMG data in the same 1-s intervals were detrended and MTA at 800 ms. The reference values of the force and EMG data detected as troughs by the algorithm were subtracted from the test values in the vicinity of the effort to account for baseline drift in either the force or EMG data throughout the trials.

The average force, zero-phase filtered and MTA at 500 ms, for each 1-s interval surrounding the center of a peak or trough was divided by the average concomitant EMG, zero-phase filtered and MTA at 800 ms, to normalize the measurements for subtle variations in measured force. At the constant forces chosen, any relative decrease in the force/EMG relationship for a device state indicates decreased electrical activity in the forearm muscle for a given effort in that device state, as, in efforts well below the individual’s maximum grip strength, the electrical activity necessary to contract a muscle linearly increases with increasing percent of maximum muscle effort [31, 35].

To analyze the data from the functional tasks, a modified version of the MATLAB algorithm was used to identify peaks and troughs in the detrended and filtered EMG data, and to extract 2 s of the EMG data with the identified peak or trough as the midpoint. To accomplish this, the processed EMG data were moving averaged over 5000 ms and zero-phase filtered before being assessed for peaks and troughs with a minimum distance between peaks set as 5000 ms. The initially processed data were extracted based on the times of the peaks and troughs as identified by this procedure. The 2 s of EMG data were, again, zero-phase filtered and MTA at 800 ms. As before, the reference values of the EMG data identified by the trough detection in the vicinity of the lifting efforts were subtracted from the peak test values to account for any baseline drift.

Results of the tests in which the subjects were bare handed, wearing the unpowered exoskeleton, and wearing the powered were compared using a one-way repeated measures ANOVA, and multiple comparisons were assessed with Fisher’s L.S.D. This analysis was performed for each of the trials described- the 25 N grasping efforts, the 15 N pinching efforts, the 8 N pinching efforts, the 15 N palmer pinching efforts, the tote bag lifting efforts, and the water bottle lifting efforts. Statistical analyses were performed using OriginPro 2018 (OriginLab) with a statistically significant different identified as p < 0.05.

## Results

During the grasping and pinching efforts that were measured by the hand-force dynamometer and as expected, there was no significant increase or decrease between the forces the subjects produced across all three testing states (p > 0.05), or in other words, the subjects produced similar forces in the trials in which they were bare handed, wearing the unpowered device, and wearing the powered device for each of the force-measured tests (25 N grasp, 15 N pinch, 8 N, pinch, and 15 N palmer pinch).

**Fig. 8:**
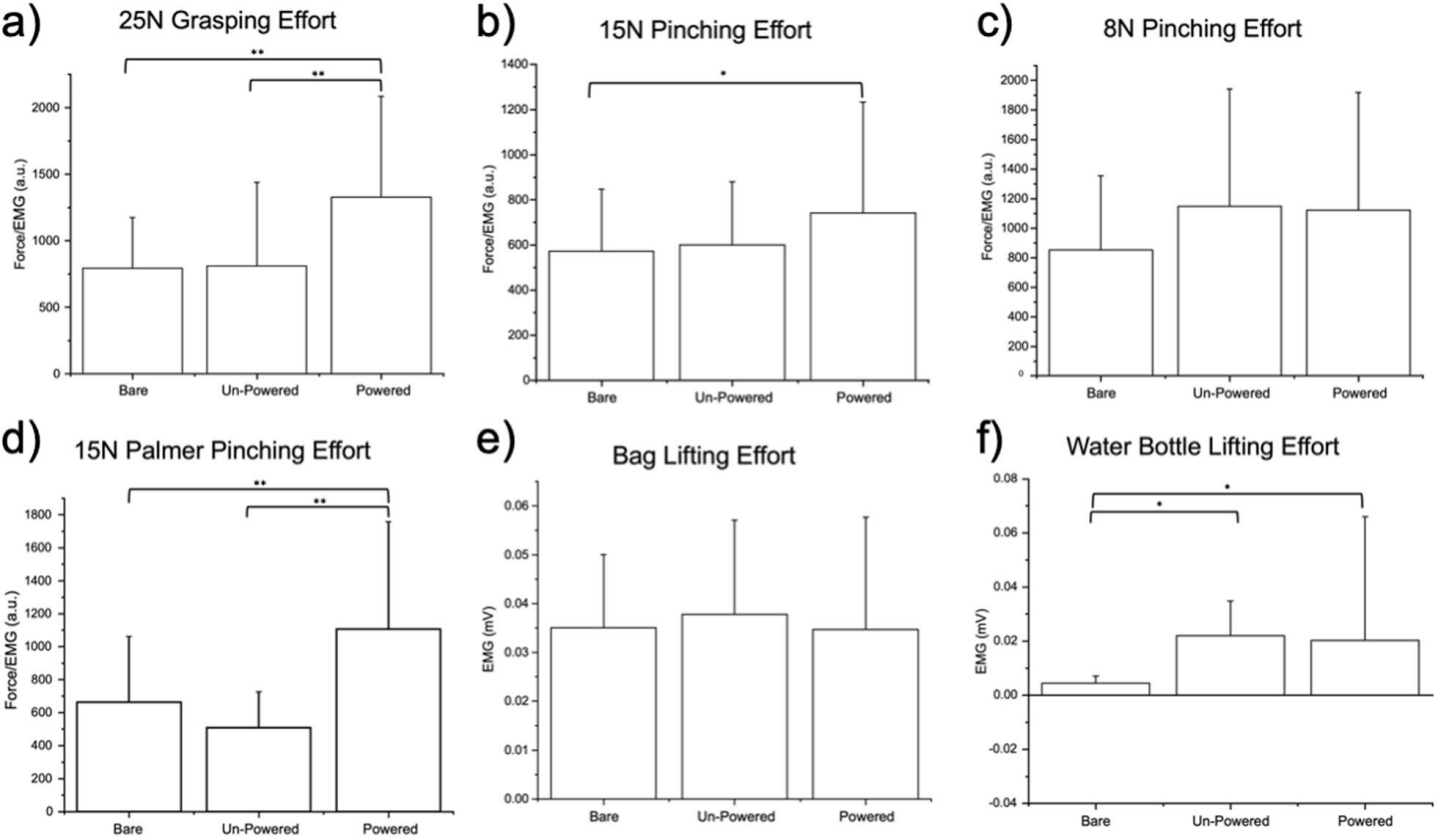
Average across 3 efforts per testing state in 10 subjects of **a** full hand grasping, **b** 15 N pinching, **c** 8 N pinching, **d** 15 N Palmer pinching force/forearm muscle EMG ratio expressed in arbitrary units (a.u.), and **e** lifting a tote bag, **f** lifting a plastic water bottle forearm muscle EMG, expressed in millivolts (mV), where error bars designate standard deviation (*p < 0.05; **p < 0.001)

**Fig. 9:**
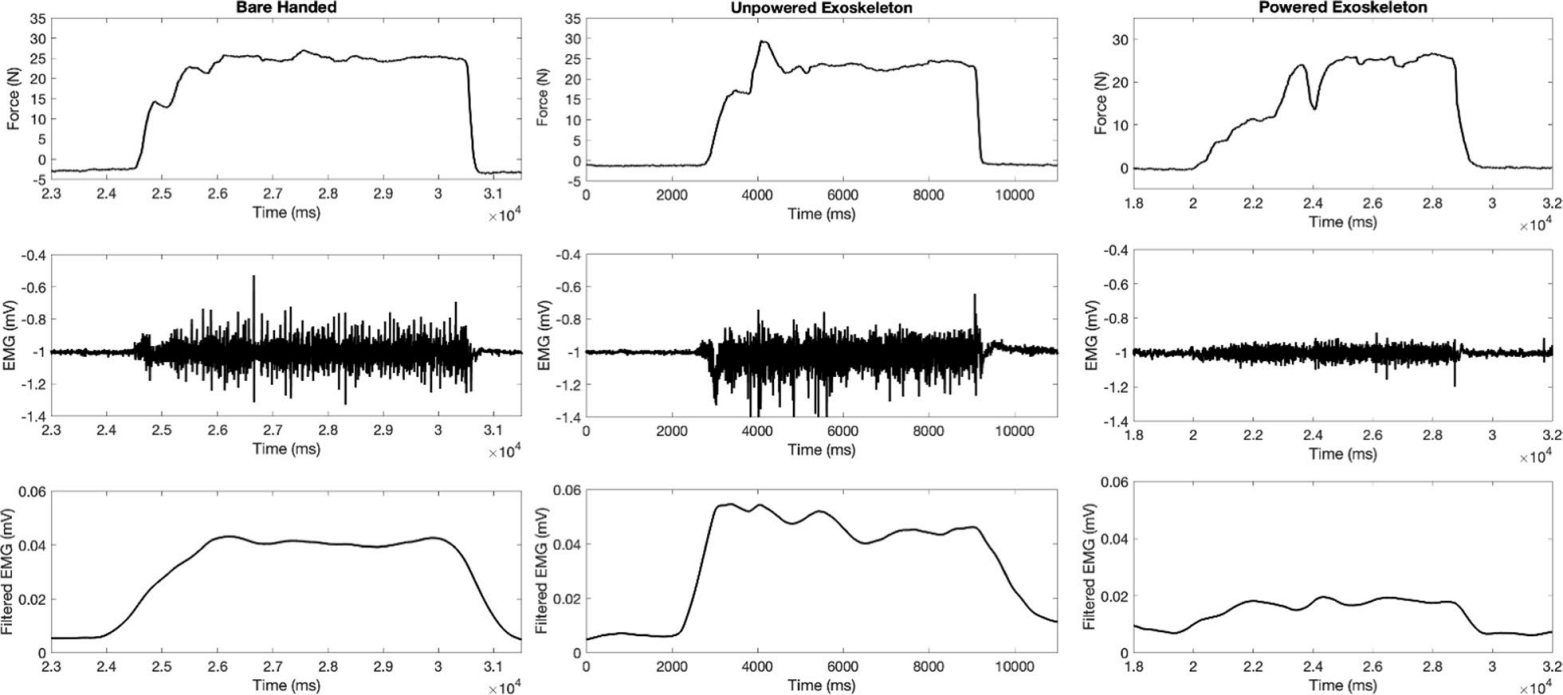
From top to bottom, recorded grasping force, recorded EMG, and detrended, filtered EMG from a representative subject during a 25 N grasping effort during all three testing states. In the columns from left to right, the subject performed grasping efforts while bare-handed, wearing the unpowered device, and while wearing the powered and functional device

During the 25 N full-handed grasping efforts, there was no statistically significant change in the force/EMG ratio comparing between the trials where subjects were barehanded and where subjects were wearing the unpowered exoskeleton structure (p > 0.05). However, there was a statistically significant (67%) increase in force/EMG ratio in the trial where subjects were wearing the powered exoskeleton device compared to the barehanded trials, meaning that there was less forearm muscle activation when the user produced the same force while wearing the powered and calibrated exoskeleton device compared to when they had no assistance (p < 0.001). This effect was also statistically significant when comparing the force/EMG ratio between the trials in which the subjects were wearing the unpowered structure and when wearing the powered device (p < 0.001). This is illustrated across the entire cohort in Fig. 8a and is also demonstrated in the data from a single subject in Fig. 9.

During 15 N thumb and forefinger pinching efforts, there was no statistically significant change in the force/EMG relationship comparing between trials where subjects were barehanded and where subjects were wearing the unpowered exoskeleton structure (p > 0.05). There was, however, a statistically significant (30%) increase in force/EMG ratio in the trial where subjects were wearing the powered exoskeleton device compared to the barehanded trials, meaning that there was less forearm muscle activation when the user produced the same force while wearing the powered and calibrated exoskeleton device compared to when they had no assistance (p < 0.05), as shown in Fig. 8b. During the 8 N pinching efforts, there was no statistically significant benefit to wearing the device. There was no statistically significant change in the force/EMG ratio where the subjects performed these light pinching efforts when using device any of the testing states (p > 0.05), shown in Fig. 8c.

During 15 N palmer pinching efforts, there was no statistically significant change in the force/EMG ratio comparing between the trials where subjects were barehanded and where subjects were wearing the unpowered exoskeleton structure (p > 0.05). There was, however, a statistically significant (67%) increase in force/EMG ratio in the trial where subjects were wearing the powered exoskeleton device compared to the barehanded trials, meaning that there was less forearm muscle activation when the user produced the same force while wearing the powered and calibrated exoskeleton device compared to when they had no assistance (p < 0.001). This effect was also statistically significant when comparing the force/EMG ratio between the trials in which the subjects were wearing the unpowered structure and when wearing the powered device (p < 0.001). This is illustrated across the entire cohort in Fig. 8d.

**Fig. 10:**
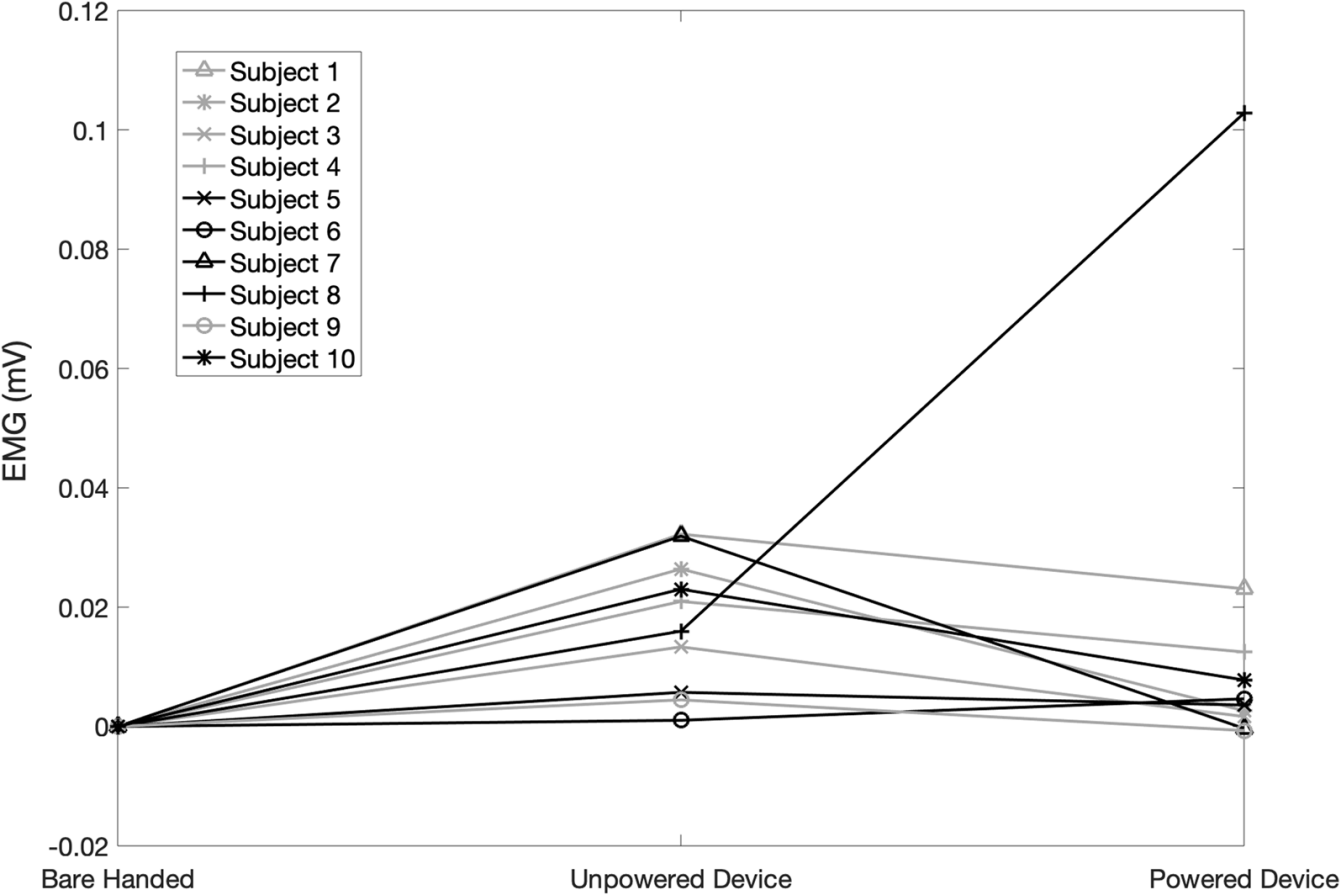
Normalized average forearm EMG (mV) from 3 efforts during the lifting of a plastic water bottle, across three states, bare handed, wearing the unpowered device, and while wearing the powered and functioning device

**Fig. 11:**
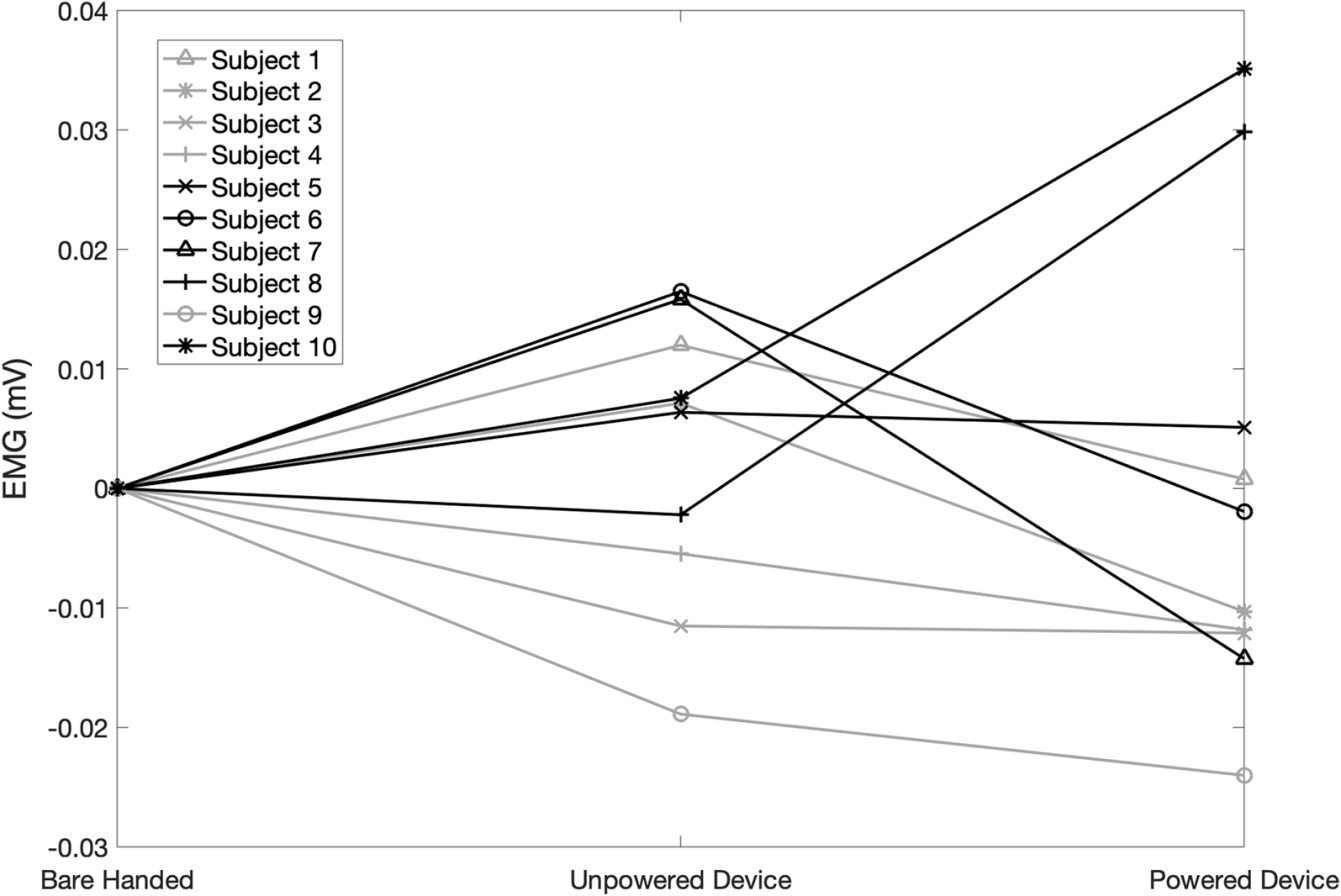
Normalized average forearm EMG (mV) from 3 efforts during the lifting of a weighted tote bag, across three states, bare handed, wearing the unpowered device, and while wearing the powered and functioning device

There were no trends attributed to wearing the device, powered or unpowered, and no statistically significant change was observed between the average EMG produced in lifting a tote bag off the floor when the subjects were barehanded, wearing the unpowered structure, and when wearing the powered device (p > 0.05), as shown in Fig. 8e. In lifting a water bottle off the table, there was a significant increase in the forearm EMG produced when the subject wore the device, either powered or unpowered, when compared to not wearing the device (p > 0.05), as shown in Fig. 8f. Therefore, the device significantly impeded lifting a small object significantly lighter than the device itself. The normalized average forearm EMG measurements across the three efforts of each individual subject during listing a plastic water bottle and lifting the weighted tote bag are shown in Figs. 10 and 11.

## Discussion

### Limitations of the design and testing protocols

Since the exoskeleton was designed to provide a rigid support, individual digit movement was constrained to concentric and eccentric trajectories in a single plane of movement. Additionally, the digits were limited to 15 degrees of freedom in order to decrease weight and cost by reducing the number of active motors necessary. In the effort to reduce weight, complexity, and cost, adduction and abduction motions of the fingers were not assisted. The entire device, including the batteries and control system, weighed approximately 0.91 kg, a potentially significant weight to be carried on the arm of an individual with any amount of reduced arm strength; however, this weight is comparable to that of other similar devices that assist motion of 5 digits, and the weight falls within the typical 0.7–5.0 kg range of other hand exoskeletons [22, 27, 36, 37].

Both Bowden cable-based and pneumatic systems require motors or compressors to generate any concentric or eccentric forces. As for soft pneumatic actuators, the artificial muscles, such as the McKibben muscle, requires air transfer conduits and a compression system. As compared to the system in our described device, neither of these ideas save weight per-se. The pneumatic system is more complex in design, and the mass of the compressor(s) and valves are usually quite significant [15, 28, 38–42]. This type of system also moves a portion of the weight and volume from the forearm to a remote pack and/or the fingers themselves [15, 28, 29, 38, 41], which also reduces the ‘realistic’ feel or likelihood of providing the ‘structural support’ to the fingers that this device aims to provide. Using a rigid exoskeleton as opposed to a soft one also increases the likelihood of the unpowered device reducing the EMG produced for a certain force as shown by some of our subjects in Figs. 10 and 11. This movement of weight and volume to fingers, or devices that do not have a ‘realistic’ feel of independent finger movement can result in increased EMG for force provided, as described in [43]. By putting the heavier components directly onto the wrist as opposed to something like a backpack or holster, we can reduce movement restrictions and perceived movement restrictions due to hanging wires, as was present in an early version of this device [23], and other devices which use Bowden-cable systems [28, 29, 38, 41].

Along with increased weight which impeded the functional test of lifting a light weight, the joints of the device provided additional friction to digit movement. Although the control system compensated for the added friction on the concentric efforts, actuator activation only applied assistive forces during these concentric efforts. Therefore, the user was required to contribute to all eccentric, or digit extension, efforts without any motor assistance, although the non-adjustable rubber bands assisted in this movement. As stated earlier, if the user were to remove the tips of their fingers from the FSRs, the exoskeleton digits would automatically extend, but would not force the extensor movement should the user resist the rubber bands, even without engaging the motors.

In order to optimize the functioning of the FSRs and linear actuator function for each subject, the lengths of the polymer cables were adjusted to best fit the subject’s combined finger and palmer lengths in order to allow for an optimal contact of the fingertips and the FSRs, and therefore maximizing the user’s interface with the control system of the device. This required the investigator to disconnect, shorten, and re-attach 5 polymer cables on the device for each subject after checking the fit of the device, but prior to recording any data. Finally, the force produced solely by the user was not recorded, the forces reported were the combination of the user’s effort and the assistive force of the device. It was determined that the separate recording of user and device force would have required additional force sensors and wiring placed inside the device that would increase the weight and friction of the joints resulting from the additional wires.

### Objective assessment of device performance

In previous studies, a grasping effort showed a statistically significant reduction in forearm muscle activation during grasping efforts for both the three-fingered [11] and five-fingered versions of this device [31]. However, in the previous pilot study with fewer participants using the five-fingered device, the 15 N pinching effort did not provide a statistically significant reduction in forearm muscle activation [31]. Now, in a sample of ten subjects with a slightly modified control scheme, while wearing the device the user needed a significantly reduced amount of forearm muscle activation for a grasping effort by 67% (Fig. 8a), a 15 N palmer pinching effort by 30% (Fig. 8d), and a 15 N pinching effort by 67% (Fig. 8b). These percent differences are comparable to reduction in EMG recorded while using a similar exoskeleton device, for either the hand or arm, whose intent is to augment a user’s force production [21, 44]. There are devices, however, that allow the user to produce minimal muscle effort, but these are device that intend to only minimally voluntary movement, where the user implies movement and the device moves semi-autonomously [15, 16].

Although there was no statistically significant increase in the force/EMG ratio in the 8 N pinching effort (Fig. 8c), the average pinching force/EMG ratio increased in both the trials where subjects wore the unpowered structure and the trials where subjects wore the powered device trials as compared to the barehanded trials (35% and 32% increase respectively). These percent differences in the 8 N pinching effort are comparable to Kadowaki, et al., where it was found that their soft exoskeleton device assisted with 20% of the pinching effort [45]. This is also comparable to the reduction in EMG produced in devices of similar structure and function, but for a different limb, for example by the major hip flexor, minor knee extensor muscle in a study investigating a powered hip exoskeleton device during walking [46]. This implies that wearing the device, powered or unpowered, provided enough support to the fingers during light pinching efforts to reduce muscle activation. It is also possible that there was reduction in activation in the other muscles of the arm and hand that were not investigated. For example, the exoskeleton also assisted in the movement of the thumb, especially in the pinching efforts, but the EMG of these muscles, such as the abductor pollicis, were not recorded. Additionally, in comparison to the HERO Grip Glove [14], this exoskeleton device itself, with no human interaction, produced 17.2 N of grip force, compared to their 12.7 N, but only 5.0 N of pinch force compared to their 11.0 N. This would appear to indicate that the thumb exoskeleton digit may be the limiting factor in the reduced pinching forces produced by this device. This is also supported by the increase in force produced when performing single/multi-finger grasps as opposed to finger-thumb pinches. These single-digit user-independent grasping forces, however, are comparable to other, similar powered assistive devices for the hand, although some with fewer digits than five, with forces ranging from 5 to 12  N per digit [20, 47, 48].

Overall EMG increased when lifting a water bottle while wearing the exoskeleton device, powered or unpowered, when compared to the trials in which the subjects were barehanded (Fig. 8f). However, the majority of subjects showed a decrease in EMG production when the device was powered on compared to when the device was unpowered, while a two saw an increase (Fig. 11).), indicating that powering the device still had a beneficial impact when grasping an object over wearing the unpowered device. This increase in EMG from bare-handed to wearing the device is likely attributed to the weight of the device, as the subjects also had to lift the device along with the water bottle in the trials in which they wore the exoskeleton device, and the weight of the water bottle was significantly smaller than the weight of the device. However, in order to reduce the weight of the device, the ability to move all five fingers independently would be lost, such as with Yoo et al. [17], where only one motor was used to control multiple fingers to decrease weight and cost. Based on some participant feedback, however, it may be possible to have one motor control both the pinky and the ring finger. Some subjects expressed that they did not feel their pinky finger contributed to their gripping capability, so having the pinky driven in parallel with ring finger motion may be a feasible method to remove some weight.

While there was no overall trend of improvement when lifting a tote bag off of the ground (Fig. 8e), most subjects showed reductions in EMG production when the device was powered as compared to their trials with the unpowered device or bare-handed, while two subjects saw an increase when comparing the powered device trials to the unpowered device trials (Fig. 10). This suggests that for some individuals, wearing the device while powered was beneficial in reducing EMG to lift certain objects, while others had a difficult time wearing or controlling the device for these purposes. For example, subject 8 had difficulty in using the powered exoskeleton for both of these tests as shown in Figs. 10 and 11.

Again, it is possible that there was reduction in activation of other muscles of the arm and hand were not investigated. In a study investigating an exoskeleton for the arm, the EMG of 16 upper limb muscles were recorded, and it was found that in different movement patterns, different muscles showed a decrease in EMG with the use of the device [49]. In the future, a more extensive array of EMG electrodes could be used to determine if different muscles of the hand and forearm showed reduction in activation during functional tests.

### Assessment of potential functionality for ADLs

Many studies assessing the functionality of novel exoskeletons for assistance in ADLs and rehabilitation assess theoretical sensory feedback [30, 50], joint torques and grip forces in controlled motion of the device [18, 36, 51], and force/EMG measurement for controlled full-hand grasps [11, 31]. This type of assessment, however, does not necessarily correlate to a device’s usefulness in performing ADLs such as lifting various objects. Although the device appeared to increase the muscle activation needed to lift an object lighter than the device itself and showed no significant increase or decrease in the muscle activation needed to lift an object heavier than the device itself; this generalization was not true on a subject-by-subject basis.

The majority of subjects saw a reduction in EMG when lifting an object heavier than the device when the device was in the powered state compared to their trials with the unpowered device or bare-handed (Fig. 11). This is likely attributed to both the subject’s grip on the object and the fit of the device. If the device was fit poorly to the individual, the subject would be more likely to lose contact with some of the FSRs in the fingertips and therefore not be fully assisted in their grip. This would also cause the subject to adjust their hand’s position in the device during the test, increasing their muscle activation. This spike in muscle activation due to repositioning would also occur when the subject adjusted their grip on the tote bag. The subjects that were more adept at controlling the device and felt more comfortable wearing it were less likely to attempt to reposition either the device or the bag, resulting in a decrease in EMG when wearing the powered device. So, while the device is not well suited to assist subjects lifting an object lighter than the device, it was beneficial when assisting subjects who felt comfortable wearing the device in lifting an object heavier than the device itself.

This would imply that the intended users of the device, or subjects who require assistance in ADLs, would require a training regimen involving repetitively using the device to complete tasks that the device would be used for. This kind of training regimen is commonly used in studies evaluating a device’s usefulness in assisting stroke patients perform ADLs [5]. Post-task-oriented training, subjects with impairments due to stroke have shown improvement in their hand functions [5], so this type of training would be beneficial for subjects who might feel uncomfortable using the device initially. In the future, subjects should undergo a task-oriented training regimen after initial grasping efforts, then their ability to perform functional tasks should be re-assessed to account for initial comfortability in using the device. Additionally, in future studies with this device, subjects with upper limb impairments should be recruited to investigate how effectively the device assists in their realistic ADLs.

### Value added

This device is a low-cost rigid exoskeleton that allows for individual finger movement and significantly reduces forearm EMG. Many force amplifying exoskeletons fit one or two of these categories, but the fact that our device fits all three is what adds value to the field. As stated previously, while wearing the device, the user required a significantly reduced amount of forearm muscle activation for a grasping effort by 67% (Fig. 8a), a 15 N palmer pinching effort by 30% (Fig. 8d), and a 15 N pinching effort by 67% (Fig. 8b), which is a comparable reduction while using a similar exoskeleton device whose intent is to augment a user’s force production [21, 44]. Although there was no statistically significant increase in the force/EMG ratio in the 8 N pinching effort (Fig. 8c), the average pinching force/EMG ratio increased in both the trials where subjects wore the unpowered structure and where subjects wore the powered device trials as compared to the barehanded trials (35% and 32% increase respectively, similar to a comparable soft exoskeleton device [45]). Even though exoskeletons with 1 or 2 fingers are simpler to implement, most ADLs require at least 3 fingers to be assisted by an exoskeleton [26]. Exoskeletons with 3 or 4 fingers could assist with most ADLs, the realism for the user would decrease as the number of fingers decrease [26]. Additionally, having fewer fingers limits the motions the user can make with their hand, as well as limit the objects the user can lift. For example, a 4 or 3-fingered exoskeleton could assist a user in picking up objects of uniform shapes (e.g., cup, reusable water bottle) but not objects that are oddly shapes or have varying thicknesses (e.g., cell phone, wine glass). Increasing the number of independently controlled exoskeleton digits would allow for the control to lift objects such as this and increase the mobility to the point where it feels natural to the user.

Using only commercially available components is only one part of making the exoskeleton cost-effective by removing the need for custom components. While this would make up some of the cost, what makes this device cost-effective is the manufacturing method. Previous designs from our laboratory were machined from aluminum, which not only resulted in a heavier exoskeleton, but also required greater time, cost, and experience to manufacture. This fabrication method also limited part and assembly design due to the inherent nature of subtractive manufacturing. By switching to thermoplastic, the device was lightened significantly, but the manufacturing method then also had to be reconsidered. Subtractive manufacturing of plastic would have largely the same problems as subtractive manufacturing of metals, and forming/molding would require an excess of 20 different molds (as each part in our device is unique to each finger) and would limit the design and assembly of the parts, as interconnected pieces cannot be formed/molded. Therefore, a main design consideration that makes the exoskeleton cost effective is the move to additive manufacture, specifically fused deposition modeling (FDM). Not only is material cost low, labor cost and time is also much lower. By using additive manufacturing, the need for expert machining, design and assembly restrictions, and the cost of developing molds was removed. This design fits into the niche of where additive manufacturing makes production cost effective, with low production volume and high complexity.

## Conclusions

In this study, the function of a newly-designed battery powered, five-fingered, 3-D printed force augmenting orthotic exoskeleton for the human hand was tested both independently and on ten healthy individuals. A control system implemented using an Arduino microcontroller proportionally commanded assistive linear actuators based on the pressure sensed by corresponding FSRs located in the distal and ventral aspect of each exoskeleton digit. The exoskeleton device allowed subjects to maintain independent control of each digit, although some of the subjects indicated that they were afraid to break the 3-D printed digits during testing. While wearing the exoskeleton, in both the unpowered and powered states, subjects were able to grasp, hold, and move common objects such as a water bottle or a bag, as well as smaller and more delicate objects, such as a smartphone or dry-erase marker.

## Data Availability

The datasets used and/or analyzed during the current study are available from the corresponding author on reasonable request.

## Abbreviations

ADLs: Activities of daily living
CAD: Computer aided design
FSR: Force sensing resistor
Ni-MH: Nickel-metal hydride
IfThP: Index finger and thumb pinch
IfG: Index finger grasp
MfG: Middle finger grasp
RfG: Ring finger grasp
HandG: Full hand grasp
EMG: Electromyography
MTA: Moving time averaged
a.u.: Arbitrary units of force/EMG
